# Antismoking Messages and Intention to Quit — 17 Countries, 2008–2011

**Published:** 2013-05-31

**Authors:** Roberta B. Caixeta, Dhirendra N. Sinha, Rula N. Khoury, James Rarick, Heba Fouad, Edouard Tursan d’Espaignet, Doug Bettcher, Linda J. Andes, Krishna Palipudi, Samira Asma

**Affiliations:** Pan American Health Organization; South-East Asia Regional Office; European Regional Office; Western Pacific Office; Eastern Mediterranean Regional Office; World Health Organization; Office of Smoking and Health, National Center for Chronic Disease Prevention and Health Promotion, CDC

Antismoking mass media campaigns can help reduce the prevalence of smoking by discouraging young persons from initiating smoking and by encouraging current smokers to quit (1,2). Smoking cessation is a multistage process; intention to quit smoking precedes quit attempts (3). To assess whether awareness of anti-cigarette smoking information in four mass media channels (television, radio, billboards, and newspapers or magazines) was significantly associated with a current cigarette smoker’s intention to quit, CDC analyzed data from 17 countries that participated in the Global Adult Tobacco Survey (GATS). Logistic regression was used to analyze the relationship between awareness of antismoking messages and intent to quit smoking; odds ratios were adjusted to control for demographic factors, awareness of warning labels on cigarette packages, and awareness of tobacco advertisements. In nine of 17 countries, intent to quit was significantly associated with awareness of antismoking messages in a single media channel versus no awareness, with adjusted odds ratios ranging from 1.3 to 1.9. In 14 countries, intent to quit was significantly associated with awareness of messages in multiple channels versus no awareness, with adjusted odds ratios ranging from 1.5 to 3.2. Antismoking information in mass media channels can help reduce tobacco consumption by encouraging smokers to contemplate quitting and might be more effective when presented in multiple channels.

GATS is an ongoing, nationally representative household survey of noninstitutionalized adults aged ≥15 years (4). This report used data from current cigarette smokers in 17 countries that participated in GATS during 2008–2011. Current smokers who were categorized as intending to quit included 1) persons who indicated they planned to quit smoking in the next month and 2) persons who indicated they were thinking about quitting smoking in the next 12 months. Survey questions asking whether current smokers noticed anti-cigarette smoking information during the last 30 days in any of four media channels (television, radio, billboards, and newspapers or magazines) were used to measure awareness of the messages.

Logistic regression was used to analyze the relationship between awareness of antismoking messages and intention to quit smoking. Awareness of antismoking messages was classified into three categories: 1) did not notice antismoking information in any media channels; 2) noticed antismoking information in one of the four channels; and 3) noticed antismoking information in more than one of the channels. Because intention to quit and exposure to antismoking information might both be associated with demographic characteristics, variables for sex, age, residence, education, and socioeconomic status (5) were entered into the model. Additionally, to control for media influence, two indicators were entered into the model: whether the respondent noticed warning labels on cigarette packages in the last 30 days and whether the respondent was aware of protobacco marketing in the last 30 days. Awareness of protobacco marketing was measured by affirmative responses to a series of questions asking whether the respondent had noticed protobacco advertisements, promotions, or sponsorships in the last 30 days in various marketing channels (6).

A total of 265,564 persons participated in the 17 country surveys. Response rates for the surveys ranged from 65.1% in Poland to 97.7% in Russia, with a median response rate of 93.6%.*

Of the participants, 50,209 reported they were current smokers.† In all 17 countries, these respondents noticed antismoking information during the last 30 days in all four of the media channels (television, radio, billboards, and newspapers or magazines). More than half of respondents noticed antismoking information in at least one of the four media channels in all countries, and more noticed antismoking information on television compared with the other three media channels. Awareness of antismoking information on television was reported by >80% of smokers in four countries: Turkey (87.8%), Malaysia (86.7%), Vietnam (85.6%), and Mexico (82.8%). Awareness for radio was highest in Mexico (47.9%), followed by Malaysia (47.0%) and Uruguay (43.0%). Awareness for billboards ranged from 17.6% in Brazil to 73.9% in Malaysia, while awareness for newspapers or magazines ranged from 9.4% in Indonesia to 74.3% in Malaysia (Table 1).

What is already known on this topic?

Mass media antismoking messages can help reduce tobacco consumption.

What is added by this report?

Among current smokers in nine of 17 countries surveyed, the association between intent to quit and awareness of antismoking messages in a single media channel versus no awareness was significant, with adjusted odds ratios ranging from 1.3 to 1.9. In 14 of the countries, the association between intent to quit and awareness of messages in multiple media channels versus no awareness was significant, with adjusted odds ratios ranging from 1.5 to 3.2.

What are the implications for public health practice?

Antismoking information in the mass media can reduce tobacco consumption by encouraging smokers to contemplate quitting and might be even more effective when presented in multiple media channels.

Among the respondents, 10,439 said they intended to quit. In five of the 17 countries, the number of respondents intending to quit was >30% (43.8% in Bangladesh, 34.6% in Mexico, 33.7% in Uruguay, 31.7% in Poland, and 30.2% in Vietnam). Intention to quit smoking was <20% in five countries (18.7% in Brazil, 16.0% in China, 14.5% in Russia, 14.2% in Malaysia, and 10.5% in Indonesia). The proportion of respondents who noticed a warning label in the last 30 days was high in all countries, ranging from 70.7% in India to 97.9% in Romania. Wide variation was observed in the percentage of respondents who noticed any type of protobacco marketing in the last 30 days, ranging from 0.0% in three countries (Egypt, Thailand, and Vietnam) where all forms of tobacco advertising, promotions, and sponsorship are banned to 87.3% in Indonesia, where only the distribution of free samples of cigarettes is banned (6,7) (Table 1).

In nine of the 17 countries, the association between intent to quit and awareness of antismoking messages in a single channel versus no awareness was significant, with adjusted odds ratios ranging from 1.3 to 1.9. The association between intent to quit and awareness of messages in multiple channels versus no awareness was significant in 14 of the 17 countries, with adjusted odds ratios ranging from 1.5 to 3.2. The strongest association (adjusted odds ratio: 3.2) was in Bangladesh (Table 2).

## Editorial Note

The World Health Organization Framework Convention on Tobacco Control requires countries to provide widely accessible, comprehensive information about the addictiveness, risks, and harms of exposure to tobacco smoke. Antismoking messages in the mass media are one means to accomplish this goal. Whereas awareness of antismoking messages demonstrates that the information has reached the public, smokers’ intentions to quit are an indicator of the effectiveness of those messages. Campaign reach, intensity, duration, and the content of messages might influence effectiveness (8).

Research has shown that mass media campaigns might be ineffective if they do not meet a threshold for sufficient population exposure. Among the GATS countries included in this study, such a threshold might be difficult to overcome without the use of television, the primary media channel associated with the greatest exposure. The content of the messages also matters; messages that convey the adverse health effects of tobacco use and secondhand smoke exposure have been found to be more effective than other message types (8).

The findings in this report are subject to at least six limitations. First, awareness of mass media antismoking messages does not directly measure the frequency or duration of exposure to specific messages. The extent of the mass media campaigns in the countries studied was not reported. Second, differences in content can be found in antismoking media messages as well as in protobacco marketing and warning labels on cigarette packages; these differences might account for differences in their association with intention to quit. Third, additional factors (e.g., increases in tobacco prices or smokefree laws) not controlled for in this analysis might influence whether smokers intend to quit (8). Fourth, different types of smoked tobacco products other than cigarettes are common in several countries (e.g., bidis in Bangladesh and India and shisha in Egypt, Turkey, and Ukraine). This report is limited to anti-cigarette smoking messages specifically and does not consider media messages aimed at the use of other types of smoked tobacco. Fifth, although intention to quit has been correlated with actual quit behavior (3,8), it is not a direct measure of quit behavior. Finally, the survey design is cross-sectional, and causality cannot be inferred from the associations described in this report.

This report adds to the body of evidence showing that awareness of mass media antismoking messages can be associated with intent to quit smoking. Mass media campaigns also can help reduce smoking prevalence by stimulating discussion and changing social norms regarding tobacco use and secondhand smoke exposure and are a crucial element of comprehensive tobacco control programs (8,9). These global findings provide additional support for CDC’s Tips from Former Smokers mass media campaign (10).

## Figures and Tables

**TABLE 1:** Prevalence of current smoking and selected characteristics of current cigarette smokers* aged ≥15 years — Global Adult Tobacco Survey, 17 countries, 2008–2011

Characteristic	Bangladesh (N = 9,629)		Brazil (N = 39,425)		China (N = 13,354)		Egypt (N = 20,924)		India (N = 69,296)		Indonesia (N = 8,305)	
					
	%	(95% CI)	%	(95% CI)	%	(95% CI)	%	(95% CI)	%	(95% CI)	%	(95% CI)
**All persons aged ≥15 yrs**												
Current cigarette smoking prevalence	14.2	(13.2–15.2)	16.9	(16.5–17.4)	27.7	(26.2–29.2)	16.3	(15.7–17.0)	5.8	(5.5–6.2)	34.8	(33.2–36.4)
**Current cigarette smokers**												
% who intend to quit†	43.8	(39.6–48.2)	18.7	(17.3–20.3)	16.0	(12.9–19.7)	27.9	(25.7–30.2)	27.8	(25.2–30.6)	10.5	(8.2–13.3)
% who noticed antismoking information in last 30 days												
On television	45.3	(40.6–50.0)	64.8	(63.2–66.3)	47.1	(41.3–52.9)	54.4	(51.7–57.1)	39.3	(36.7–41.9)	38.8	(34.6–43.2)
On radio	16.8	(13.4–20.8)	32.3	(30.8–33.8)	6.3	(4.2–9.2)	17.1	(15.3–19.1)	17.9	(15.9–20.0)	5.5	(4.3–7.0)
On billboards	22.6	(19.4–26.3)	17.6	(16.3–18.8)	20.8	(16.8–25.5)	28.3	(25.9–30.9)	25.2	(23.0–27.5)	32.1	(28.2–36.3)
In newspapers or magazines	12.8	(10.8–15.2)	36.3	(34.7–37.9)	22.3	(18.5–26.6)	16.0	(14.5–17.8)	32.7	(30.2–35.3)	9.4	(7.8–11.3)
In any of the above four media channels	55.1	(50.2–59.9)	72.0	(70.5–73.4)	56.7	(50.7–62.6)	65.3	(62.8–67.8)	58.6	(55.8–61.4)	51.6	(47.0–56.1)
% who noticed warning labels on cigarette packaging	90.7	(88.5–92.6)	87.9	(86.8–88.9)	87.5	(82.9–91.0)	98.6	(97.9–99.0)	70.7	(68.0–73.2)	72.2	(67.4–76.6)
% who noticed protobacco marketing	54.9	(50.0–59.6)	37.7	(36.0–39.3)	17.3	(14.1–21.1)	0.0§	—	18.8	(16.8–21.1)	87.3	(84.9–89.4)

Characteristic	Malaysia (N = 4,250)		Mexico (N = 13,617)		Philippines (N = 9,701)		Poland (N = 7,840)		Romania (N = 4,517)		Russia (N = 11,406)	
					
	%	(95% CI)	%	(95% CI)	%	(95% CI)	%	(95% CI)	%	(95% CI)	%	(95% CI)
**All persons aged ≥15 yrs**												
Current cigarette smoking prevalence	22.9	(21.0–25.0)	15.6	(14.5–16.8)	27.9	(26.8–29.2)	30.2	(28.8–31.5)	26.7	(25.0–28.4)	38.8	(37.4–40.2)
**Current cigarette smokers**												
% who intend to quit†	14.2	(10.9–18.3)	34.6	(31.5–37.9)	20.7	(18.6–23.0)	31.7	(29.2–34.3)	23.5	(20.9–26.4)	14.5	(12.7–16.4)
% who noticed antismoking information in last 30 days												
On television	86.7	(83.0–89.8)	82.8	(80.4–84.9)	57.1	(54.1–60.0)	58.2	(55.1–61.2)	75.1	(71.2–78.6)	38.2	(35.6–40.9)
On radio	47.0	(42.1–52.0)	47.9	(44.2–51.5)	40.3	(37.5–43.2)	26.9	(24.5–29.4)	22.3	(19.6–25.4)	10.1	(8.7–11.8)
On billboards	73.9	(70.0–77.6)	36.3	(33.6–39.2)	23.9	(21.5–26.5)	23.5	(21.2–26.0)	29.8	(26.7–33.1)	27.8	(24.8–31.0)
In newspapers or magazines	74.3	(69.8–78.4)	51.6	(48.1–55.1)	29.4	(26.9–32.1)	37.4	(34.5–40.3)	35.2	(32.2–38.4)	31.7	(29.4–34.2)
In any of the above four media channels	91.2	(87.7–93.8)	89.6	(87.9–91.2)	70.2	(67.4–72.8)	67.7	(64.6–70.6)	81.1	(77.4–84.2)	60.8	(57.8–63.7)
% who noticed warning labels on cigarette packaging	93.0	(90.3–94.9)	84.5	(82.2–86.5)	89.1	(87.1–90.8)	96.7	(95.6–97.4)	97.9	(96.5–98.8)	94.5	(93.0–95.7)
% who noticed protobacco marketing	4.6	(2.6–7.9)	53.7	(50.5–56.9)	66.3	(63.4–69.2)	20.0	(17.8–22.3)	48.6	(45.0–52.3)	70.1	(67.5–72.6)

Characteristic	Thailand (N = 20,566)		Turkey (N = 9,030)		Ukraine (N = 8,158)		Uruguay (N = 5,581)		Vietnam (N = 9,925)	
				
	%	(95% CI)	%	(95% CI)	%	(95% CI)	%	(95% CI)	%	(95% CI)
**All persons aged ≥15 yrs**										
Current cigarette smoking prevalence	23.5	(22.6–24.5)	31.1	(29.9–32.5)	28.6	(27.5–29.8)	24.7	(23.1–26.4)	19.9	(18.7–21.1)
**Current cigarette smokers**										
% who intend to quit†	24.1	(22.0–26.3)	27.8	(25.5–30.2)	25.9	(23.6–28.3)	33.7	(30.4–37.1)	30.2	(27.2–33.3)
% who noticed antismoking information in last 30 days										
On television	71.6	(69.1–73.9)	87.8	(85.8–89.5)	44.6	(41.4–47.9)	68.3	(64.6–71.7)	85.6	(82.4–88.3)
On radio	32.3	(29.7–34.9)	24.9	(22.3–27.6)	10.6	(9.2–12.1)	43.0	(38.8–47.3)	27.1	(24.6–29.7)
On billboards	37.4	(34.6–40.2)	43.9	(40.6–47.3)	26.4	(23.5–29.5)	52.4	(49.0–55.9)	41.2	(38.2–44.4)
In newspapers or magazines	21.8	(20.1–23.7)	53.7	(50.7–56.6)	26.5	(24.1–29.0)	33.9	(30.7–37.1)	31.1	(28.2–34.1)
In any of the above four media channels	81.2	(79.1–83.2)	91.4	(89.7–92.8)	57.5	(54.3–60.6)	82.3	(79.3–85.0)	89.7	(86.6–92.1)
% who noticed warning labels on cigarette packaging	93.4	(92.2–94.5)	94.5	(93.1–95.7)	96.4	(95.1–97.4)	96.4	(94.7–97.5)	95.0	(93.5–96.2)
% who noticed protobacco marketing	0.0§	—	3.8	(3.0–5.0)	37.4	(34.4–40.4)	21.2	(18.2–24.5)	0.0§	—

**Abbreviation:** CI = confidence interval.

* Current cigarette smokers included those who smoked manufactured cigarettes, handrolled cigarettes, or kreteks, daily or less frequently than daily.

† Current smokers who were categorized as intending to quit included 1) persons who indicated they planned to quit smoking in the next month and 2) persons who indicated they were thinking about quitting smoking in the next 12 months.

§ All forms of tobacco advertising, promotions, and sponsorship are banned in Egypt, Thailand, and Vietnam.

**TABLE 2:** Odds ratios for current cigarette smokers* who intend to quit†, by awareness of anti-cigarette smoking information — Global Adult Tobacco Survey, 17 countries, 2008–2011

Awareness of anti-cigarette smoking information	Unadjusted OR		OR adjusted by demographic variables§		OR adjusted by demographic variables and noticing warning labels		OR adjusted by demographic variables, noticing warning labels, and noticing protobacco marketing	
			
	OR	(95% CI)	AOR	(95% CI)	AOR	(95% CI)	AOR	(95% CI)
**Bangladesh**								
Did not notice anti-cigarette smoking information	1.0		1.0		1.0		1.0	
Noticed anti-cigarette smoking information in one channel	1.3	(0.9–1.8)¶	1.3	(0.9–1.9)¶	1.3	(0.9–2.0)¶	1.4	(0.9–2.1)¶
Noticed anti-cigarette smoking information in multiple channels	2.8	(1.9–4.1)	2.9	(1.9–4.4)	3.0	(2.0–4.5)	3.2	(2.1–4.8)
**Brazil**								
Did not notice anti-cigarette smoking information	1.0		1.0		1.0		1.0	
Noticed anti-cigarette smoking information in one channel	1.6	(1.2–2.1)	1.7	(1.3–2.2)	1.6	(1.2–2.1)	1.6	(1.2–2.1)
Noticed anti-cigarette smoking information in multiple channels	2.0	(1.6–2.5)	2.1	(1.6–2.6)	1.9	(1.5–2.4)	2.0	(1.6–2.5)
**China**								
Did not notice anti-cigarette smoking information	1.0		1.0		1.0		1.0	
Noticed anti-cigarette smoking information in one channel	1.5	(1.0–2.1)	1.6	(1.1–2.2)	1.6	(1.1–2.3)	1.6	(1.1–2.2)
Noticed anti-cigarette smoking information in multiple channels	1.7	(1.2–2.4)	2.0	(1.4–2.9)	2.1	(1.5–3.0)	2.1	(1.5–3.0)
**Egypt**								
Did not notice anti-cigarette smoking information	1.0		1.0		1.0		1.0	
Noticed anti-cigarette smoking information in one channel	1.1	(0.8–1.3)¶	1.1	(0.8–1.4)¶	1.1	(0.8–1.4)¶	1.1	(0.8–1.4)¶
Noticed anti-cigarette smoking information in multiple channels	1.7	(1.3–2.2)	1.6	(1.3–2.1)	1.6	(1.3–2.1)	1.6	(1.3–2.1)
**India**								
Did not notice anti-cigarette smoking information	1.0		1.0		1.0		1.0	
Noticed anti-cigarette smoking information in one channel	1.7	(1.2–2.2)	1.8	(1.3–2.4)	1.7	(1.3–2.4)	1.8	(1.3–2.4)
Noticed anti-cigarette smoking information in multiple channels	1.9	(1.5–2.5)	2.1	(1.6–2.8)	2.1	(1.6–2.8)	2.1	(1.6–2.8)
**Indonesia**								
Did not notice anti-cigarette smoking information	1.0		1.0		1.0		1.0	
Noticed anti-cigarette smoking information in one channel	1.8	(1.2–2.7)	1.8	(1.2–2.8)	1.7	(1.1–2.6)	1.9	(1.2–3.1)
Noticed anti-cigarette smoking information in multiple channels	1.8	(1.2–2.8)	1.8	(1.2–2.7)	1.7	(1.1–2.6)	1.9	(1.2–3.0)
**Malaysia**								
Did not notice anti-cigarette smoking information	1.0		1.0		1.0		1.0	
Noticed anti-cigarette smoking information in one channel	0.3	(0.1–1.2)¶	0.4	(0.1–1.4)¶	0.4	(0.1–1.3)¶	0.4	(0.1–1.3)¶
Noticed anti-cigarette smoking information in multiple channels	1.1	(0.4–3.1)¶	1.2	(0.4–3.7)¶	1.1	(0.4–3.2)¶	1.1	(0.4–3.0)¶
**Mexico**								
Did not notice anti-cigarette smoking information	1.0		1.0		1.0		1.0	
Noticed anti-cigarette smoking information in one channel	1.1	(0.7–1.7)¶	1.0	(0.7–1.7)¶	1.0	(0.6–1.6)¶	1.0	(0.6–1.6)¶
Noticed anti-cigarette smoking information in multiple channels	1.4	(1.0–2.1)¶	1.4	(0.9–2.1)¶	1.4	(0.9–2.1)¶	1.3	(0.9–2.0)¶
**Philippines**								
Did not notice anti-cigarette smoking information	1.0		1.0		1.0		1.0	
Noticed anti-cigarette smoking information in one channel	1.8	(1.2–2.5)	1.7	(1.2–2.4)	1.7	(1.2–2.4)	1.7	(1.2–2.4)
Noticed anti-cigarette smoking information in multiple channels	1.9	(1.4–2.6)	1.7	(1.2–2.3)	1.7	(1.2–2.3)	1.7	(1.2–2.3)
**Poland**								
Did not notice anti-cigarette smoking information	1.0		1.0		1.0		1.0	
Noticed anti-cigarette smoking information in one channel	1.9	(1.4–2.6)	1.9	(1.4–2.7)	1.9	(1.4–2.6)	1.9	(1.4–2.6)
Noticed anti-cigarette smoking information in multiple channels	2.0	(1.5–2.7)	2.1	(1.6–2.7)	2.0	(1.5–2.7)	2.0	(1.5–2.6)
**Romania**								
Did not notice anti-cigarette smoking information	1.0		1.0		1.0		1.0	
Noticed anti-cigarette smoking information in one channel	1.8	(1.1–2.9)	1.8	(1.1–3.0)	1.9	(1.1–3.0)	1.9	(1.1–3.1)
Noticed anti-cigarette smoking information in multiple channels	2.4	(1.5–3.7)	2.4	(1.5–3.7)	2.4	(1.5–3.7)	2.4	(1.5–3.7)
**Russia**								
Did not notice anti-cigarette smoking information	1.0		1.0		1.0		1.0	
Noticed anti-cigarette smoking information in one channel	1.4	(1.1–1.9)	1.4	(1.0–1.8)	1.4	(1.0–1.8)	1.3	(1.0–1.7)
Noticed anti-cigarette smoking information in multiple channels	1.8	(1.3–2.5)	1.8	(1.3–2.4)	1.8	(1.3–2.4)	1.7	(1.3–2.3)
**Thailand**								
Did not notice anti-cigarette smoking information	1.0		1.0		1.0		1.0	
Noticed anti-cigarette smoking information in one channel	1.6	(1.2–2.1)	1.6	(1.2–2.1)	1.5	(1.1–2.1)	1.5	(1.1–2.1)
Noticed anti-cigarette smoking information in multiple channels	2.1	(1.5–2.8)	2.0	(1.5–2.7)	2.0	(1.4–2.7)	2.0	(1.4–2.7)
**Turkey**								
Did not notice anti-cigarette smoking information	1.0		1.0		1.0		1.0	
Noticed anti-cigarette smoking information in one channel	1.4	(0.9–2.2)¶	1.4	(0.9–2.2)¶	1.4	(0.9–2.2)¶	1.4	(0.9–2.2)¶
Noticed anti-cigarette smoking information in multiple channels	1.5	(1.0–2.3)	1.5	(1.0–2.3)	1.5	(1.0–2.3)	1.5	(1.0–2.3)
**Ukraine**								
Did not notice anti-cigarette smoking information	1.0		1.0		1.0		1.0	
Noticed anti-cigarette smoking information in one channel	1.3	(1.0–1.8)¶	1.2	(0.9–1.7)¶	1.2	(0.9–1.7)¶	1.2	(0.9–1.7)¶
Noticed anti-cigarette smoking information in multiple channels	2.0	(1.5–2.7)	1.8	(1.3–2.4)	1.8	(1.3–2.4)	1.8	(1.3–2.4)
**Uruguay**								
Did not notice anti-cigarette smoking information	1.0		1.0		1.0		1.0	
Noticed anti-cigarette smoking information in one channel	1.0	(0.6–1.7)¶	1.0	(0.6–1.8)¶	1.0	(0.6–1.8)¶	1.0	(0.6–1.8)¶
Noticed anti-cigarette smoking information in multiple channels	1.2	(0.8–1.8)¶	1.2	(0.8–1.7)¶	1.2	(0.8–1.7)¶	1.2	(0.8–1.7)¶
**Vietnam**								
Did not notice anti-cigarette smoking information	1.0		1.0		1.0		1.0	
Noticed anti-cigarette smoking information in one channel	1.9	(1.0–3.7)	2.1	(1.0–4.1)	2.0	(1.0–4.0)¶	2.0	(1.0–4.0)¶
Noticed anti-cigarette smoking information in multiple channels	2.8	(1.5–5.2)	2.9	(1.5–5.7)	2.8	(1.4–5.5)	2.8	(1.4–5.5)

**Abbreviations:** OR = odds ratio; AOR = adjusted odds ratio; CI = confidence interval.

* Current cigarette smokers included those who smoked manufactured cigarettes, handrolled cigarettes, or kreteks, daily or less frequently than daily.

† Current smokers who were categorized as intending to quit included 1) persons who indicated they planned to quit smoking in the next month and 2) persons who indicated they were thinking about quitting smoking in the next 12 months.

§ Demographic variables were sex, urban/rural residence, age group, education, and socioeconomic status. Data for Brazil were not adjusted for education because measures of education were not comparable with the other countries.

¶ The association between intent to quit smoking and awareness of anti-cigarette smoking information was not significant (p≥0.05).

## Footnotes

* Response rates for the 17 country surveys were as follows: Bangladesh 93.6%, Brazil 94.0%, China 96.0%, Egypt 97.2%, India 91.8%, Indonesia 94.3%, Malaysia 85.3%, Mexico 82.5%, Philippines 94.7%, Poland 65.1%, Romania 89.1%, Russia 97.7%, Thailand 94.2%, Turkey 90.9%, Ukraine 76.1%, Uruguay 95.2%, Vietnam 92.7%.

† Current cigarette smokers included those who smoked manufactured cigarettes, hand-rolled cigarettes, or kreteks, daily or less frequently than daily.
